# Modern Sociology

**Published:** 1900-04-14

**Authors:** 


### Modern Sociology.

### THE DRILL SERGEANT IN THE BOARD SCHOOL.

The circular issued by Sir Henry Craik, the secretary of the Scotch Education Department, at the recommendation of Lord Balfour of Burleigh, on the subject of physical education in schools, is peculiarly *àpropos* just now. It requires some courage to quote again the Duke of Wellington's saying that his battles were won on the playground at Eton, when the Eton boys themselves are so tired of the constant repetition of it; but it is the weak point of all truths that, to indent them into the public mind, they have to be repeated to weariness. But Eton and similar schools provide only for the physical training of the leaders, and it is only in comparatively recent times that anything has been done to secure the bodily development of the future private soldier. Yet for him special physical education is far more necessary than for the Etonian, whose circumstances generally make healthful, pleasurable exercise easily attainable. As Lord Balfour says, physical training, especially military drill, "not only tends to improve manual dexterity and to render more alert the faculties of observation, but is also pre-eminently useful in developing those habits of comradeship, of responsibility, and of individual resource which are of supreme importance, not only to the nation as a whole, but to the individual pupil."

It cannot be denied that Lord Balfour is right in combining the importance to the individual with the importance to the nation, of good health in all its sons. A man who is not physically "fit" is as much handicapped in the race of life as one who cannot read and write. That which is easy to other men is difficult to him. Moreover, things which are attainable to him through his intellectual powers are forbidden to him by his physical limitations. In proof of this, witness those brilliant students who pass at the top for the Indian Civil Service, only to be rejected by the doctor, as unable to stand the climate. Less conspicuously, perhaps, but as surely, the same thing goes through all other spheres of life. Thus we come back to the old notion, that the sound mind must be placed in a sound body if it is to fulfil its utmost usefulness to its possessor and to his country. And, as Lord Balfour says, "Attention to physical training becomes all the more urgent owing to the tendency of population to gather to the larger towns, where the opportunities for physical exercises are necessarily restricted," The country boy plays cricket, or football, or hockey, not perhaps with complete attention to the science of the game, but at least with abundant physical energy; but what can we do for the boy of the town?

In the first place, give him as good a playground as we can. But, alas! the town boy sometimes does not know how to play—to play in riotous healthy, manly fashion, that is. He must be taught to exercise his muscles. In most of our large schools there is some instruction in drill, given perhaps by the janitor, or by a drill instructor engaged by the School Board. The janitor is preferable, if he is an old soldier or sailor, because he is always on the spot. There is a general and growing preference for military or naval men for such posts, and, by way of making the services more attractive to our youths, it would be well if such posts should be, as far as possible, reserved for those who have served the Queen. Apart from the national duty of encouraging men to enter the army and navy by holding out to them the prospect of decently remunerative employment for them after they leave the service, these men, who have learned the value of promptness, accuracy, and obedience, and who have besides that general "handiness" which the average man lacks, are by far the best fitted for a post of such general utility. And with an old soldier on the premises drill might be made a more regular feature of education than it can be when it depends on the visits of an itinerant drill instructor. It is true that our climate does not always permit of outdoor instruction of any kind, but many large schools are now made with a central hall, where drill can be taught at any time, and the provision of such a hall should be made a feature of all school architecture in the future.

When the playground alone is available, there must be more eagerness in seizing the shining hour. That is, the drill lesson must no longer take place on a fixed hour of a fixed day of the week, and if that particular hour prove unfavourable, be given up. If Monday be rainy, let some indoor lesson be given, so that an hour may be free for drill on Tuesday, if that day should prove fine. It must be recognised that physical training may be quite as useful for the school-boy in after life as Latin or mathematics. The head master must learn that the physical lesson is not a waste of time, but an essential part of education. In time, he will find that boys who have had their lungs expanded and their muscles stretched by drill, come back to book work with renewed energy. The teachers in the infant-room know that, but those of the upper classes have been more slow to grasp it. Children who have got languid and drowsy at sedentary work are freshened by a little physical exercise. It may be said that play gives them this, but they may be too fagged to desire to play. Drill is compulsory play, and gives that physical energy which we associate with our games. Boys, and girls too, for that matter, will be more fit for play as well as for study after half an hour with the drill master.

As we hope that the boy who has learned to read and write will not in after life lose these accomplishments, but rather put them to good use, so we may reasonably hope that the boy who has gone through a course of elementary drill at school will not become lazy and inert, but will rather incline to take up some form of amusement that will continue the exercise. Boys thus trained will want to join cadet corps when they leave school; they will join a volunteer regiment when they get older; they will be, to a greater extent than our youth have hitherto been, citizen soldiers. Seeing that Britons, who "never shall be slaves," have a rooted objection to conscription, although they are as brave and patriotic as any nation, it seems likely that some such method of developing and training the instinctive military spirit of youth will be the simplest and most natural method of increasing our army. Many men would join the regular troops who now never think of it, if once they had acquired a love for military exercises; and those who did not would be able, at a pinch, to fight for their country better than they can do now. Above all, they would have a respect for the army which would react healthily on the self-respect both of the army and the nation.

